# Hemofiltration in Patients with Severe Acute Pancreatitis (Review)

**DOI:** 10.17691/stm2020.12.1.14

**Published:** 2020

**Authors:** G.A. Boyarinov, P.S. Zubeyev, K.V. Mokrov, O.V. Voyennov

**Affiliations:** Professor, Head of the Department of Anesthesiology and Resuscitation, Privolzhsky Research Medical University, 10/1 Minin and Pozharsky Square, Nizhny Novgorod, 603005, Russia; Professor, Head of the Department of Emergency Medical Care, Privolzhsky Research Medical University, 10/1 Minin and Pozharsky Square, Nizhny Novgorod, 603005, Russia; Head of the Resuscitation and Anesthesiology Unit, City Hospital No.33, 54 Lenin Avenue, Nizhny Novgorod, 603076, Russia; Professor, Department of Anesthesiology and Resuscitation, Privolzhsky Research Medical University, 10/1 Minin and Pozharsky Square, Nizhny Novgorod, 603005, Russia

**Keywords:** severe acute pancreatitis, endogenous intoxication, hemofiltration

## Abstract

Questions regarding the application of extracorporeal detoxification to patients with severe acute pancreatitis have been considered. Hemodialysis, the historically first method of extracorporeal detoxification for such patients, has been also described in the review. Appropriateness of using renal replacement therapy methods and among them continued renal replacement therapy has been shown.

Hemofiltration and hemodiafiltration technologies are described in detail including different modes of their application and the possibility of using various types of filters. Available data on hemofiltration for patients with severe acute pancreatitis have been analyzed.

Great attention is paid to the unsolved aspects of hemofiltration in severe acute pancreatitis such as determining renal and extrarenal indices; time of starting hemofiltration; selection of volume replacement modes and a buffer system; procedure duration; anticoagulation measures, defining criteria to assess the adequacy of hemofiltration, state severity, and organ dysfunction degree.

Further multicenter investigations are necessary to be able to assess the efficacy of the hemofiltration procedures on the basis of the thoroughly worked out and pathogenically grounded protocol using adequate control methods taking into consideration endogenic intoxication phases and intensity of the multiple organ failure syndrome.

## Introduction

Acute pancreatitis (AP) is a primary aseptic inflammation of the pancreatic gland which may result in the damage of the surrounding tissues as well as distal organs and systems [1]. Until now, severe acute pancreatitis (SAP) remains a disease with a high risk of unfavorable outcome [2–4].

The mortality rate in patients with SAP reaches 20–60% depending on the disease character, phase of the pathological process, severity of the comorbid pathology, intensity of the multiple organ failure syndrome (MOFS), purulent-septic complications, and septic shock development [4–7]. The main factors determining a poor prognosis are the rate of MOFS and sepsis development and their intensity as well as septic shock [2, 4, 6, 8]. In case of MOFS occurrence and progression in the first 48 h, we speak of early severe pancreatitis which is characterized to a larger extent by unfavorable prognosis [1, 3]. Unsatisfactory results of treatment of this patient cohort, especially those at the active working age, and high economic cost determine socio-economic significance of the disease and importance of improving diagnostic and treatment methods [1, 2].

Extracorporeal detoxification technologies along with other methods are commonly used for intensive therapy of patients with SAP. A special place among these methods is occupied by hemofiltration (HF) aimed at eliminating toxic substances with middle- and low-molecular weight from the blood by convection [1, 4, 9–11].

## Appropriateness of using hemofiltration in patients with severe acute pancreatitis

In AP, tissues of retroperitoneal space are involved in autoenzymatic necrobiosis, necrosis, and postnecrotic infection [4]. To determine the severity of AP patients, a modified Atlanta classification suggested by the International Association of Pancreatology (2011) and classification by Acute Pancreatitis Classification Working Group (2012) are used. According to these classifications, patients with AP of moderate severity are observed to have one of the local complications: acute parapancreatic fluid collection, acute necrotic collection and/or persistent organ failure lasting up to 48 h. A severe course of AP is characterized by obligatory presence of local complications and development of persistent organ failure continuing more than 48 h [1, 3, 4]. Thus, AP is said to be severe in case of MOFS formation and the difference between a moderate and severe forms in organ failure may be connected only with the temporal factor.

Diagnosing of AP is based on the assessment of the following criteria: clinical picture, characteristic signs according to US and CT data, hyperenzymemia (3 times and more) [1, 4, 12–14].

Acute pancreatitis is differentiated into edematous and necrotic [1, 4]. The incidence rate of edematous (interstitial) pancreatitis accounts for 80–85%. It is characterized by a mild severity and rare development of local complications or systemic disorders [1, 15, 16].

Necrotic pancreatitis (pancreonecrosis) occurs in 15–20% of patients, clinically always manifests itself as a moderate or severe disease, has a phase course with two peaks of lethality: early and late. The early phase lasts usually for the first two weeks, the second (late) phase up to several months [1, 4]. The early phase, in its turn, is subdivided into two periods.

Phase IA is the first week of the disease. In this period, formation of necrotic foci of various size in the pancreatic parenchyma or surrounding cellular tissue and the development of endotoxicosis take place. Endotoxicosis manifests itself by systemic disorders in the form of organ (multiple organ) failure of different intensity. Necrosis is usually formed in the pancreas during 3 days maximally, in severe pancreatitis, the period of its formation is much shorter (as a rule 24–36 h). Accumulation of enzymatic effusion is going on in the abdominal cavity (enzymatic peritonitis and parapancreatitis) — one of the sources of endotoxicosis. Transient dysfunction of separate organs and systems is observed in moderate disease severity. Phenomena of organ (multiple organ) failure: cardiovascular, respiratory, renal, hepatic, etc., may prevail in the clinical picture of severe forms of pancreatitis [1, 4].

Phase IB occurs in the second week of the disease. It is characterized by a body response to the formed necrotic foci (both in the pancreas and parapancreatic cellular tissue). Clinically, phenomena of resorptive fever prevail, peripancreatic infiltrate is being formed [1, 14, 15, 17, 18].

Phase II is a late sequestration phase, which usually begins from the third week and may continue for several months. Sequesters in the pancreas and postperitonial cellular tissue are commonly formed beginning from the fourteenth day of the disease onset. When large sequesters are rejected, fistulous tracts appear [1, 14–18]. Two variants of this phase are possible. Aseptic sequestration, sterile pancreonecrosis, is characterized by isolated fluid collection in the pancreas region and postnecrotic pseudocysts of the pancreatic gland. Septic sequestration occurs in case of infection of the pancreas parenchyma necrosis and parapancreatic cellular tissue with the development of purulent complications. The clinical form of this phase is infection pancreonecrosis which may be circumscribed (abscess) or non-circumscribed (purulent-necrotic parapancreatitis). When purulent complications are progressing, infection pancreonecrosis may have its own complications (purulent-necrotic leakages, abscesses of the postperitoneal space and abdominal cavity, suppurative peritonitis, erosive and gastrointestinal bleedings, digestive fistulas, sepsis, etc.) with the development of endotoxicosis of infection genesis, organ (multiple organ) failure [1, 2, 4, 8, 14, 16–18].

SOFA score is used to assess organ and multiple organ dysfunction. To determine the severity of patient’s state and impossibility of using multiparametric scales, it is recommended to apply clinical and laboratory criteria: signs of the systemic inflammatory response syndrome; hypocalcemia <1.2 mmol/L; hemoconcentration (hemoglobin >160 g/L or hematocrit >40 units, hyperglycemia >10 mmol/L); C-reactive protein >120 mg/L; shock (systolic BP<90 mm Hg); respiratory failure (рО_2_<60 mm Hg); renal failure (oligoanuria, creatinine >177 μmol/L); hepatic insufficiency (hyperenzymemia); cerebral insufficiency (delirium, sopor, coma); gastrointestinal bleeding (>500 ml/day); coagulopathy (thrombocytes <100·109/L, fibrinogen <1.0 g/L) [1].

MOFS pathogenesis in SAP is connected with immune system activation and release of inflammatory mediators (tumor necrosis factor, interleukins IL-1, IL-6, IL-8, IL-10, platelet-activating factor, and others [1, 4, 9, 19, 20]. The emerging disorders of vascular tone regulation and changes in the vascular wall permeability result in the impairment of organ perfusion, in particular, mesenteric, pulmonary, and cerebral blood supply [1, 4, 21]. There occurs massive sequestration of fluid to the interstitial space, postperitoneum cellular tissue, abdominal and pleural cavities with the development of heavy hypovolemia and electrolyte disorders in combination with the collection of excessive substances with a toxic effect in the liquid body media causing metabolic disturbance at the cellular and subcellular levels and, ultimately, mitochondrial dysfunction [20, 22].

Local inflammatory process and impairment of intestinal mucosa microcirculation, developing and progressive intestinal paresis contribute to translocation of bacterial microflora and the occurrence of endotoxemia triggering an entire pool of metabolic changes [22–27].

One of the key moments in the transformation of the AP clinical picture into its severe course is the transfer of local inflammatory processes to the systemic inflammatory response. Historically, the comprehension of this process occurred at the beginning of the XXI century when a concept of interaction of systemic inflammatory response and multiple organ dysfunction was formulated [20–22, 28]. The mechanism of multiple organ dysfunction development consisting of 5 stages was proposed.

*The first stage (induction)* represents a local inflammatory reaction in the area of traumatic impact or in the infection focus. At this stage, interactions of multiple mediators are directed towards the limitation of the damaging agent spreading. A compensatory anti-inflammatory reaction defends the organism against autodestruction.

*The second stage (cascade)* is formation of minor foci of the secondary damage to the organs and tissues and primarily in the area of the capillary bed due to the started endothelial dysfunction.

*The third stage (secondary autoagression)* implies a release of the secondary inflammatory mediators due to alteration of immunocompetent cells and modulation of specific protein synthesis transcription. This phase corresponds to the systemic inflammatory response syndrome (SIRS). Aggravation of the process ends by the development of the acute blood circulation insufficiency according to the mechanism of distributive shock.

*The fourth phase (compensatory anti-inflammatory response syndrome)*, CARS, represents triggering of the remaining compensatory capabilities at all adaptation levels (subcellular, cellular, tissue, organ, systemic).

*The fifth stage (terminal)* develops in case of compensatory processes failure and is completed with organism death [20, 23, 29, 30].

In recent years, a term CHAOS was introduced to describe the predominant phase of the pathological process in the clinical picture: C stands for cardiovascular dysfunction, SIRS prevails; H means homeostasis impairment, multidirected effects of SIRS and CARS; A denotes apoptosis, SIRS and CARS are suppressed; O is organ dysfunction, SIRS; S is immunosuppression, CARS. CHAOS phase is finished by multiple organ damage, MOFS, increasing significantly the risk of death for patients with sepsis [20, 23, 24, 30, 31].

Severe AP is one of the most frequent causes of abdominal sepsis [1, 4, 20]. Now, sepsis is understood to be life-threatening organ dysfunction caused by a dysregulated response of the organism to infection, a heavy state with the development of organ failure caused by the infection process [20, 23, 29–31].

Sepsis is known to be a critical state associated with endogenous intoxication which causes mitochondrial dysfunction, triggers the mechanisms of apoptosis and immunodepression [19, 22, 29–31]. The complex of these pathogenic mechanisms is maintained and regulated by the mediators of systemic inflammation, coagulation, reprogramming of immunocompetent cell genes, and disorders of physiological functions and organ activity in this case go beyond the limits of regulatory capabilities to such an extent that the function regulation systems are not able to correct spontaneously and demand partial or complete replacement (prosthetic treatment) [31–36].

Septic shock is an extreme and the most prominent manifestation of sepsis and is characterized by MOFS decompensation with the involvement of all functional systems of homeostasis support in the pathological process, with progressive critical tissue hypoperfusion [22, 29–32]. Some investigations have convincingly shown that complex water-sectoral disturbances develop in septic shock: excess of the total fluid is formed in presence of deficit of the circulating blood volume associated with the fluid redistribution from the vascular sector to the interstitial and intracellular space [29–32].

Heavy sepsis and septic shock are characterized by marked hypoxia and multiple organ failure maximal manifestations of which are registered in hypodynamic type of blood circulation [19, 21, 22, 29, 30, 32].

Signs of endotoxicosis syndrome manifest themselves concurrently with the development of generalized inflammatory processes being the phenomenon of uncontrolled effect of a plurality of toxic substances of endogenous origin on the organism under the conditions of insufficiently functioning detoxification systems [19, 22, 32–35]. It is the intensity of intoxication that determines in many respects the severity of patient’s state and the degree of organ dysfunction [1, 4, 19, 22, 32]. The success of severe generalized infection treatment depends in many ways on the efficacy of therapeutic measures directed to the primary infection focus and poisoning substance [1, 4, 19].

The mechanism of endotoxicosis development may be associated with the metabolism disorder (metabolic), difficulty of elimination (retentional), absorption of toxic substrates into the blood flow (resorptive) [34]. Endotoxicosis may also be caused by the effect of various toxic compounds on the body [1, 4, 22, 32, 35]. The most characteristic of them are products of microflora life activity: exo- and endotoxins, effector immunological substances, neuromediators of the body regulatory systems, biogenic amines, thyroid and steroid hormones, antibodies, immune complexes, prostaglandins, factors of coagulation and fibrinolytic systems [20, 24, 25, 32, 34]. More than 30 cytokines alone can be distinguished: interleukins, interferons, colony-stimulating factors, tumor necrosis factors, compounds transforming growth and differentiation of monocytes, complement system [29, 34, 35, 37].

Intoxication can be maintained by the formation of supermolecular biological compounds between the microorganism elements and products of the native tissue decomposition [32, 34–36].

Uncontrollable inflow of substances with enzymatic activity from the damaged organs such as trypsin, lipases, lysosomal enzymes also contribute to autoagression [32, 33–35].

Increased concentration of end or intermediate products of normal metabolism, pathological shifts in electrolyte balance also cause toxic effect [34, 36].

Carbon dioxide, lactate, pyruvate, urea, creatinine, uric acid, aromatic amino acids, ammonium salts, bilirubin, non-esterified fatty acids, sodium, potassium, calcium acquire the property of poisons in case of pathological change in the tissue metabolism processes [34]. Their accumulation is possible in case of retentional mechanism realization, when elimination processes are impaired, and when the excessively generated amount of them enter the blood [34].

Substances from circumscribed cavity fluid media of the body, for example, intestine are also toxic [32, 34, 35].

Penetration of phenol, indole, cadaverine, alcohols, aldehydes, ketones, carboxylic acids into the bloodstream results in a general toxic effect [32, 34, 35].

Thus, organ hypoperfusion, tissue hypoxia, endotoxicosis, systemic inflammatory and anti-inflammatory responses induced by “mediator aggression”, water-sectoral and electrolyte disorders are the most important links of etiopathogenesis of SAP and MOFS associated with it [1–4, 7, 9, 21, 31, 34, 35, 38–40]. Consequently, application of detoxification methods directed to the removal of toxic substances, normalization of water-sectoral and electrolyte disturbances, normalization of organ perfusion, decrease of systemic inflammation phenomena while treating patients with SAP is pathogenetically justified in order to prevent MOFS and the development of pancreatic shock and sepsis [1–4, 24, 25, 34, 35, 38–42].

## Hemofiltration and hemodiafiltration in severe acute pancreatitis

Hemofiltration, one of the extracorporeal detoxification methods, is used in the renal replacement therapy (RRT). For this purpose, blood from the vascular catheters is collected to the extracorporeal contour with anticoagulant and passes across a semipermeable membrane of the hemofilter which filtrates plasma under hydrostatic pressure gradient cutting off the molecules with small and middle molecular weight to the removable exfusate [43–46]. HF stands close to the glomerular filtration in terms of molecular spectrum of removable substances and uremic toxins [46–51]. Replacement of water-electrolyte substances is realized in the pre-and postdilution modes with influent solutions [52–54]. This method was proposed by L.V. Genderson et al. in 1967 and, owing to the improvements of the filtering membranes and equipment, it has gained a wide application in clinical practice as one of the main RRT methods [54–56].

The first experience of applying extracorporeal methods of detoxification in patients with severe pancreatitis (pancreonecrosis) was described in 70–80s of the ХХ century. Those were hemodialysis procedures for acute kidney failure (AKF) [55, 56]. Soon, other methods started to be applied. Alongside with plasmapheresis and hemoperfusion to correct homeostasis, HF and hemodiafiltration (HDF) came into use [57–60]. And already by 1997, a sufficiently large experience has been gained in using different methods of extracorporeal detoxification. HF and HDF appeared to be the most representative methods with regard to the effective blood purification from toxins in patients of intensive therapy units [55, 61–64]. In case of sepsis, application of continuous veno-venous hemofiltration (CVVH) is necessary for elimination of toxic substrates and hemocorrection aimed to overcome MOFS and prevent septic shock [65–67]. Presently, CVVH is considered not only as a RRT method in acute renal injury but also as a pathogenic method of treating sepsis, septic shock, hepatorenal syndrome, SAP according to the so-called extrarenal indications [4, 34, 44, 54, 55, 62, 67–70].

The HF mechanism differs essentially from hemodialysis. Blood purification by HF is carried out by means of convection transport of substances dissolved in plasma across a filtration membrane under the action of the transmembrane pressure. Clearance, or blood purification from the substances with molecular mass up to 20,000 Da, depends on the filtration rate and duration. The feasibility of ultrafiltration rate regulation allows for the water balance control, while an application of replacing electrolyte solutions helps maintain the water-electrolyte balance [34, 54, 56].

Special highly permeable membranes from polyacrylonitrile, polymethyl methacrylate, polysulfone or cellulose-triacetate are used in hemofilters. Hemofilters used in the clinics provide 40–200 ml of hemofiltrate per minute depending on designation. Hemofilter capacity is determined by porosity and membrane area, transmembrane pressure, blood flow rate, hematocrit, and plasma protein content [34, 54]. When blood flow rate increases, blood movement in the hemofilter grows as well but concentration of polarizing substances on the membrane diminishes and the filtration flow increases. Considering the concentration polarization phenomenon, it is recommended to use a relatively high blood flow rate (250–300 ml/min) and maintain the pressure in the hemofilter of about 0.22 bar [34, 54, 56, 66, 67].

Replacement of the removed filtrate with the infusion of a special solution is also an important process simulating tubular reabsorption. The solution composition must be close to the protein-free part of plasma, have normal osmolarity and pH, possess correcting ability. A HF session requires 18–25 L of solution: 1 L for filling and air removal from the system and 0.5 L for blood return when disconnected [34, 54, 71, 72].

Influent infusion volume is determined by the necessity to remove excess water from the organism and BP control. To avoid hemodynamic disorders, the entire volume of pure ultrafiltrate is collected gradually during the procedure. Generally, the liquid removal rate must be precisely balanced with replacement. Influent is added to blood before hemofiltration (predilution) and after it (postdilution). Predelution provides a lower value of hematocrit in the hemofilter but requires more solution [34, 54, 72].

Both low and middle molecular weight toxins are eliminated during HF but low molecular weight toxins are removed worse than during routine hemodialysis. Therefore, HDF method has been proposed in which circulation of dialyzing solution in the hemodialysis filter is added to the above-described procedure providing the diffusion process [34, 51, 56, 68, 72, 73]. Application of the dialyzing solution for CVVH is not required making this method more cost-effective. The authors [74] report similar ability of HF and HDF to eliminate cytokines. In the works devoted to CVVH for patients with multiple organ failure, there was noted reduced concentration of complement components, elimination of uremic toxins, cytokines, lipopolysaccharides, normalization of blood acid-base composition, the possibility to correct electrolyte and liquid balance, improvement of detoxification liver properties in adults and children [46, 68, 69, 71, 72]. Organ-protective effects in the development of acute pulmonary injury, cardiovascular insufficiency, and encephalopathy are also described [55, 68, 71, 72, 75–80]. Some publications demonstrate the increased survival rate in case of early application of CVVH in patients with acute renal damage [44, 51, 69, 70, 81]. An important and incontestable advantage of continuous RRT performed by HF method is the possibility to effectively monitor volemic balance for a long time even under the conditions of massive infusion therapy and maintain electrolyte and acid-base balance [34, 56, 72, 81, 82]. The employment of isotonic ultrafiltration with substitution of exfusate with officinal polyionic solutions containing bicarbonate and electrolyte cations in compliance with normal indices for human plasma contributes to it to the greater extent [34, 56, 72].

After the publication of the next work by R. Bellomo et al. in 1993, the application of the veno-venous scheme via a double-lumen large-diameter catheter became a commonly accepted and preferable practice [69–71].

Hemofiltration implies usage of the latest polymer membrane filters making it possible to influence the mass transfer of biologically active substances across the membrane depending on the ultrafiltration rate, positive transmembrane gradient, and membrane sieving coefficient [34, 56, 72]. A technical feasibility of transmembrane pressure changes during the procedure provides the possibility to influence differentially the clearance of the middle molecular-weight substances (proteins and supramolecular protein structures) and large molecules [34, 56]. The higher the transmembrane pressure, filtration rate, and sieving coefficient the better is elimination of large molecules and their amount from the blood [34, 56, 81].

The main factors limiting this process are the ability of vascular access to provide a satisfactory blood flow and the characteristics of the filtering system [34, 56]. At the same time increase of the filtration rate results in uncontrollable hypercoagulation due to the growth of hematocrit in the output tract of the filtering element and adsorption of the protein compounds on the membrane surface including fibrinogen with its subsequent activation [34, 56, 82].

To solve this problem, it is reasonable to use predilution method [34, 56, 63, 72, 83]. It allows for a marked increase of filtration rate without the increase of blood flow volume [34, 70, 72, 73]. In this case, a significant reduction of endogenous toxin concentration takes place in the blood passing through the filter, but upon the whole, the improvement of toxic substances clearance is possible by sieving coefficient increase [34, 56, 70, 83].

It has been established that the majority of current filters for HF are capable of deposing active complement components, tumor necrosis factor, IL-6, IL-10, endotoxins of gram-negative bacteria on its surface [58, 62, 63, 74, 84–88]. Membranes from polymethyl methacrylate supplement the filtration clearance to the largest extent by the ability to stabilize the absorbed substances on its surface. To the least extent, the sorption activity is characteristic of polysulfone and polyacrylonitrile at the similar sieving coefficient [34, 56].

By the present time, three main hypotheses have been proposed explaining positive therapeutic effects of HF application. Immunomodulation hypothesis (Honoré concept) explains positive effects not only by the reduction of the peak concentrations of toxic substances in the blood but also by immunologic reaction change, suppression of hypermetabolism and hyperinflammation [66, 89].

Hypothesis of peak concentrations (Ronco concept) links the improvement of patients’ state directly with the drop of toxic substances in the blood [68–71].

The hypothesis of mediator delivery postulates the possibility of diffuse drift of water-soluble particles associated with inflammation from the intercellular space due to manifold increase of lymphatic drain related to high doses of postdilution (3–5 L/h) into the intravascular space with their following elimination during HF [72, 90, 91].

HF and HDF acquire special significance when natural detoxification mechanisms became impaired providing, at the same time, elimination of biologically active substances and metabolic products replacing the functions of physiological detoxification systems and primarily those of kidneys [4, 34, 49, 53, 71, 74]. For this reason, renal and extrarenal indications are distinguished for their beginning [4, 34, 52, 56, 68, 69, 74, 81]. Extrarenal indications are necessary for homeostasis correction when renal functions are preserved, while renal indications imply impaired kidney functions [4, 34, 52, 56, 68, 69, 81]. Therefore, there are different recommendations on the procedure initiation and specificity of its performance [4, 34, 72, 81]. In 2000, Ronco and Bellomo [92] formulated the following indications for RRT initiation to which they referred renal (nonobstructive oliguria/anuria, life-threatening electrolyte disturbances, metabolic acidosis, volume overload, progressive azotemia, clinical manifestations of uremia) and extrarenal (septic shock, acute pulmonary injury/acute respiratory distress syndrome or its high risk requiring massive hemotransfusion, provision of infusion therapy and nutritive support, acute cerebral injury with brain edema, chronic heart failure with diuretic-refractory edemas, rhabdomyolysis, severe burns, AP, heavy dysnatriemia, exogenous intoxications, malignant hyperthermia).

Later, Mehta [93] presented general criteria of RRT initiation for critically ill patients having divided them into the procedures for prevention of irreversible organ injuries (heart, lungs, brain) and those for removal of the consequences of “mediator burst” (severe sepsis, pancreatitis, acute respiratory distress syndrome). Thus, the rationale of using RRT as the procedure for multiple organ support has been grounded.

Later on, this idea was supported and further developed in MOST concept [71] according to which HF and HDF are considered as methods for managing multiple organ failure in the intensive therapy unit, and here early symptoms and the spectrum of organ dysfunctions as well as the main background as the factor of further MOFS progression are taken into account rather than the diagnosis [54, 55, 81, 91]. Consequently, more importance is now attached not so much to the diagnosis as to the early signs and intensity of organ dysfunctions [55, 81, 91, 94].

Conformity with this thesis allowed for determining the trend in HF and HDF application in earlier periods of critical state development [68, 73, 77, 91]. But at the same time, there have been disagreements until now on the criteria of initiation of different RRT methods. For example, in the National Manual on Intensive Therapy (2018), the indications for its performance are divided into absolute and relative. Absolute indications are as follows: plasma urea more than 36 mmol/L, uremic encephalopathy, pericarditis, neuro- and myopathy, hyperkaliemia >6.5 mmol/L, hypermagnesemia >4 mmol/L, acidosis, pH<7.15, oligoanuria <200 ml per 12 h or anuria, volume overload, brain edema, pulmonary edema, exogenous poisoning with dialyzed poisons, acute kidney injury stage III. Relative indication for RRT includes acute kidney injury stage II. Sepsis, SAP, acute respiratory distress syndrome, heavy combined trauma, hepatorenal syndrome, cardiosurgery, rhabdomyolysis were referred to extrarenal indications [4].

According to Mukhoedova [81], indications for RRT are hyperkaliemia >6.5 mmol/L, blood plasma creatinine — 250–300 μmol/L, urea — 22–25 mmol/L, hypernatremia >150 mmol/L, hypervolemia with a threat of pulmonary or brain edema resistant to diuretics, decompensated metabolic acidosis (рН<7.2; ВЕ>–8 mmol/L) intractable to conservative correction, oliguria (diuresis less than 0.5 ml/kg/h) associated with hypovolemia correction >6–12 h, anuria.

At present, the role and indications for initiation of HF and HDF in patients with SAP remain rather disputable [1, 3, 4, 8, 95]. On the one hand, these procedures are not recommended for routine practice, on the other, the development of SAP implies the occurrence of endogenous intoxication and MOFS and this is a ground for using HF and HDF procedures [4, 9–11, 63, 65, 75, 81, 91].

To improve the results of treating patients with SAP, other methods are being developed: operation technologies, application of epidural analgesia and some pharmaceuticals, nutritional support, and infusion therapy [1, 3, 4, 8, 95–100].

But at the same time, the role of endotoxicosis in MOFS development in SAP should not be ignored since it is associated with a poor prognosis [101]. Data from the recent publications should also be taken into account. They demonstrate that methods of extracorporeal detoxification, including HF, are effective for endotoxicosis management in patients with SAP that is signified by the reduced intensity of systemic inflammatory response and lower complication rate [57, 102–106]. Positive effect of HF in intestine barrier dysfunction caused by destructive pancreatitis is related to the improvement of the cytokine status owing to the removal of proinflammatory cytokines and antioxidant stress inhibition [107, 108].

However, it should be kept in mind that physiological substances requiring replacement are also eliminated from the organism together with the pathogenic matters. It may be related to the membrane structure and properties. This issue requires additional investigations [109].

The majority of investigators engaged in this problem indicate to the usefulness of assessing the efficacy of the procedure conducted to eliminate pathological substances from the patient blood. Some authors recommend not only to study the dynamics of cytokines but also to evaluate the efficacy of HF by changes in the inflammation markers such as C-reactive protein, presepsin, procalcitonin, middle molecules [34, 40, 48, 56, 59–62, 75, 81, 85, 91, 105, 106].

Khoroshilov et al. [59] have found in their investigation that the level of aromatic phenylcarbonic acid in the blood serum of patients with organ dysfunction severity by the SOFA scale was above 10 points which was significantly greater than the norm. The HDF procedure resulted in 1.5–2-fold reduction of serum concentration of these acids. This allowed the authors to make a conclusion on the possibility of using the method of assessing clearance of aromatic microbial metabolites (p-hydroxyphenylacetic and p-hydroxyphenyl lactic acid) as biomarkers for studying the effectiveness of extracorporeal detoxification methods.

An interesting feature of HF is its ability to remove and bind endotoxin. This fact is very important as the endotoxin triggers and accelerates activation and release of the majority of inflammation mediators [25–27, 34, 38, 48, 56, 58, 73–75, 79, 81, 87]. Endotoxin elimination during HF is most likely to occur due to convection and adsorption on the hemofilter membranes [56, 63, 65, 79, 82, 87, 88]. Investigations in this direction may be rather perspective. According to the data obtained by Rodnikov et al. [109], HF in the complex therapy of pancreonecrosis influences positively the condition of cellular and humoral immunity which is manifested by the increase of IgG content by 300%, IgM — 200%, IgA — 11%, Т-lymphocytes — 20%, and phagocytes — 48.6% relative to the initial data. The authors have also defined the HF effect on the coagulation system which is signified by the decrease of fibrinogen amount by 17.4% on the fifth treatment day, thrombocytes by 21.6%, prothrombin index by 28.5%. Besides, data are presented on the beneficial results of the combined use of HF and sodium hypochlorite.

At present, a question related to the dose of substitute replacement in critically ill patients during HF and HDF is being actively discussed [38, 47, 52, 56, 67, 73, 105, 106, 110]. It has been previously shown that HF dose increase to 35 ml/kg/h allows the lethality rate to be reduced by 20%, on average, in patients with severe sepsis by the elimination of freely circulating cytokines fraction which blocks further development of the pathological process. But a wide application of the proposed method in septic shock revealed patients who appeared to be resistant to homeostasis stabilization [67, 68]. Soon, Honoré et al. [66, 67] showed that increase in the replacement volume to 6.0 L/h for 6–8 h improves significantly 28-day survival in the examined group of patients. Besides, there were patients in whom marked changes in the mediator level were not determined that does not fully fit into the “hypothesis of peak concentrations”.

Currently, three main HF strategies which are gaining a worldwide spread have been formulated.

*The first* is a continuous treatment with an ultrafiltration dose (replacement volume) of 35 ml/kg/h for 24–72 h, continuous veno-venous hemofiltration (CVVH) [70].

*The second* is a continuous treatment with an ultrafiltration dose of 50–70 ml/kg/h for 24–72 h, continuous high-volume hemofiltration (СHVHF) [66, 67].

*The third* is called an intermittent high-volume hemofiltration (IHVHF) with a replacement volume up to 100–120 ml/kg/h for 6–8 h (previously called a pulsе high-volume hemofiltration, pulsе HVHF) [111, 112].

Currently, researches are being carried out to compare the efficacy of various RRT methods operating at different replacement rate. The results are rather controversial. Thus, according to many authors, CVVH application was useful in patients with critical state including those with AP since it improved the course of the disease, eliminated toxic substrates, stabilized hemodynamics, reduced lethality [34, 38, 44, 46, 75, 77, 91, 109, 113–119]. But at the same time, some investigators did not demonstrate convincing advantages of CVVH in patients with sepsis, septic shock, SAP, and MOFS [53, 67, 73, 120–122].

Moreover, the numerous data demonstrated advantages of СHVHF which enabled improvements of gas exchange, hemodynamics, cytokine elimination more effectively compared to CVVH [52, 53, 67, 73, 105, 106, 110, 122–129]. Besides, there were no significant differences found in the IVOIRE investigation [130] in 28-day lethality, severity of organ dysfunctions, improvement of hemodynamics between the groups with sepsis and acute kidney injury which underwent continuous RRT in the doses of 70 and 35 ml/h. Similarly, in the HEROICS study, no advantages of high-volume HF were also shown [131].

In the work by Kudryavtsev et al. [72, 73], better indices of lethality were noted when using IHVHF. The authors compared the treatment results of 24 patients undergone IHVHF with replacement volume of 100 ml/kg/h for 4 h and 22 patients treated with CHVHF with the filtration dose of 50 ml/kg/h and 48 h duration. The study has shown that in the IHVHF group, 28-day lethality was 29.2% which was significantly different from the CHVHF group (40.9%). In the course of the work, contraindications to IHVHF in patients with the body mass index (BMI) over 25 have been defined. Based on the data obtained, the authors have concluded that IHVHF is indicated for patients with septic shock and BMI<25. If BMI is >25, the obligatory condition for this procedure is maintaining the blood flow rate within 320–350 ml/min that provides filtration fraction not more than 25% and prevents hemofilter clotting. CHVHF is indicated to patients with heavy sepsis, prominent organ dysfunction with the score above 5.3 according to SOFA.

The investigators [53] have shown the advantages of high-volume HF in comparison with the classic one; they determined that patients with septic shock needed inotropic support with lower doses of noradrenaline if this procedure is used.

However, high-volume HF procedures are connected with the risk of rebound syndrome development and require, among other things, the control of intracranial pressure and the level of consciousness [132]. HF duration is of great importance. It has been established, that HF duration for 24–72 h enables elimination of water-sectoral and hemodynamic disorders in septic shock and diminishes the laboratory manifestations of endotoxicosis and hypoxia [34, 56, 69, 73, 75, 83, 105, 115, 116, 133]. Attempts were undertaken to objectivize criteria for early HF beginning. For example, in severe abdominal sepsis and septic shock, the necessity of early beginning of continuous RRT, in the authors’ opinion [38], is justified since it makes it possible to compensate rapidly endogenous intoxication, relieve hypoxia, make multiple organ failure less prominent, and ultimately to improve the disease outcome: lethality rate fall by 11.6% relative to the late beginning of the continuous RRT and by 28.2% in comparison with the common intensive treatment. The best results were noted in patients with hyperdynamic type of blood circulation and the worst with hypodynamic circulation. In their work, the authors recommend to start HF as early as possible not waiting for the transformation of hemodynamics into hypodynamic circulation.

Solving the question of initiation of continuous RRT procedure, the stage of the pathological process (subcompensation or decompensation), as well as the MOFS intensity, should be taken into consideration. In particular, this dependence in abdominal sepsis was found by Shukevich [38]. He showed that in sepsis under conditions of compensated variant of endogenous intoxication and absence of hypoxia (lactate concentration does not exceed 1.7±0.2 mmol/L) multiple organ failure does not develop (score less than 1.4±0.3 according to SOFA). In case of subcompensation variant of endogenous intoxication (values of endogenous intoxication syndrome index grow from 11.8±0.8 tо 15.1±0.6 RU), hypoxia develops leading to formation and progression of MOFS (the score increases from 7.3±0.5 tо 10.8±0.7 according to SOFA). In the decompensated variant (values of endogenous intoxication syndrome index reach 24.6±2.6 RU) and intensive hypoxia, multiple organ failure aggravates rapidly being most severe in hypodynamic type of septic shock (the score grows from 15.6±0.3 tо 19.5±0.5 according to SOFA).

In heavy sepsis, continuous RRT in the HF mode allows for compensation of endogenous intoxication (values of endogenous intoxication syndrome index become lower from 18.2±3.6 tо 3.8±0.7 RU), hypoxia arrest (lactate concentration reduces from 3.2±0.8 tо 1.5±0.3 mmol/L), decrease of the multiple organ failure intensity (the score falls from 12.6±0.5 tо 5.6±0.5 according to SOFA), and the lethality reduction to 27.8% in comparison with 44.4% typical for the standard intensive treatment. Continuous RRT in septic shock is able to compensate endogenous intoxication (values of endogenous intoxication syndrome index go down from 21.3±3.2 tо 3.9±1.1 RU in a hyperdynamic variant of septic shock and from 23.4±3.6 tо 5.1±1.8 RU in hypodynamic shock). The intensity of hypoxia also decreases (lactate concentration declines from 3.6±1.2 tо 1.3±0.8 mmol/L in a hyperdynamic variant of septic shock and from 3.3±0.8 tо 2.1±0.7 in hypodynamic shock) as well as that of multiple organ failure (the score falls from 15.4±0.5 tо 5.7±0.3 according to SOFA in the hyperdynamic variant of septic shock and from 16.5±0.6 tо 9.5±0.8 in the hypodynamic variant). Besides, continuous RRT decreases lethality to 38.1% relative to 57.1% typical for the standard intensive treatment in patients with the hyperdynamic variant of septic shock and to 72.7% relative to 80% in patients with the hypodynamic type of circulation [38].

In recent years, there appeared a rising interest to assess perspectives of early start of continuous RRT, i.e. prior to a full-scale clinical and laboratory picture of MOFS in order to prevent its development and progression. Until now there have been different opinions on the criteria of early beginning of continuous RRT in patients with MOFS of different etiology [4, 34, 38, 55, 71, 73, 77, 81, 91, 105, 109, 113, 116, 117, 121, 133]. On the one hand, application of continuous RRT before MOFS involves high economic expenditures related to the procedure. On the other hand, this procedure will not always ensure a positive result in case of purulent-septic complications with prominent MOFS. The medicine faces a dilemma whether to begin the procedure a bit earlier and spend money with a great probability of saving life but with the benefit which is not less than in treatment without HF, or to use this method later, when indications are evident and expenditures are grounded in terms of evidence-based medicine but with potentially worse prognosis. The available data demonstrate usefulness of an early start of continuous RRT mainly only in AKF [4, 38, 70, 121, 123, 126]. However, in recent times more and more adherents of MOST concept consider continuous RRT as a method for MOFS treatment and prevention rather than a method for treating AKF [55, 71, 106]. Many authors believe that the decision on the initiation of this procedure must be made rapidly [4, 56, 77, 81, 113, 116, 117]. It has been shown that for patients with septic shock optimal results are observed if the procedure of high-volume HF for preventing multiple organ failure was started within the first 6–12 h of admission [73]. But it remains unclear whether these data can be interpolated to the patients with SAP without shock.

The National Guidelines on Intensive Care [4] recommends starting RRT for patients with SAP in case of AKF or extrarenal indications in the first 24 h of admission to the intensive care unit. For example, Aleksandrova et al. [133] believe that the results of diagnosing early SAP on admission may serve as the criteria for the beginning of continuous RRT. They are: the state severity according to the APACHE II classification system score >12, Ranson criteria >5, SOFA score >4, failure of more than 2 organs.

Mukhoedova [81] thinks that CVVH, CVVHDF in the dose of 20–35 ml/kg/h and IHVHF in the dose of 4–6 L/min within 1–15 days may be recommended for patients with SAP. In any case, grounds for initiation of continuous RRT appear if there is organ dysfunction, MOFS, or shock [4, 81]. However, according to the data of Zhang et al. [121], early routine application of CVVH would be ineffective for patients with SAP. At the same time, other authors [11] advocate early involvement of extracorporeal methods of detoxification for patients with pancreatitis emphasizing that early inclusion of extracorporeal detoxification into the complex of treatment of destructive pancreatitis results in the decrease of clinical signs of endotoxicosis by 30–50%, accelerates normalization of hemodynamics, and reduces lethality.

Duration of CVVH procedure is also the subject of discussions. The majority of the researchers recommend determining the HF duration individually depending on the state severity. The general principle is: the more severe the patient’s state, the longer lasts the procedure [38, 54, 56, 68, 133]. According to the National Guidelines on Intensive Care [4], RRT procedure should be discontinued when the diuresis rate is restored to 400 ml/day though this recommendation needs discussion. It is important that none of the sources shows clear advantage of one RRT method over another. When comparing intermittent and continuous HF methods, the majority of the investigators gave preference to the latter in multiple organ failure with purulent-septic complications of the abdominal organ diseases [4, 111]. It has the following parameters: replacement volume not less than 45–50 L, duration not less than 24 h at the blood flow rate over 150 ml/min and individual choice of water balance mode. To assess the efficacy and safety, it is recommended to monitor effective albumin concentration, reserve albumin binding capacity as well as the dynamics of integral indices of endotoxicosis severity assessment: intoxication coefficient and water sector condition [34, 56, 68, 73, 91, 115].

The solution used to replace the removed filtrate in HF is of great importance. Currently, balanced bicarbonate solutions are employed to correct water-electrolyte and acid-base disorders in pre- and postdilution modes. The latter makes the procedure more economic, while the former usually requires prolonged time of effective filter operation [4, 56, 134].

Unfractinated heparin or sodium citrate is used for anticoagulation [4, 38, 56, 73, 91, 133, 135, 136].

In recent times, many researchers pay attention to the technological characteristics of the filters possessing the properties of sorbents [137–139]. Criteria of the efficacy of the continuous RRT method used to treat patients with SAP are very important. Various assessment scales such as APACHE II, SOFA, SAPS are mainly used [6, 38, 44, 51, 55, 73, 140, 141]. The most informative indices reflecting the severity of patient condition in pancreatitis on admission are the data of APACHE II, Ranson, SOFA scales, the number of the damaged organs, as well as BMI and patient’s age. To predict the disease outcome, the following parameters should be taken into consideration: high blood urea level, hyperglycemia, metabolic acidosis, reduction of the respiratory index, severity of organ failure according to the SOFA score. Patients with SAP with progressive multiple organ failure (early SAP) are referred to the group of risk of unfavorable outcome at the early phase of the disease. Application of continuous CVVH with complex intensive therapy is indicated to this group of patients [133].

To evaluate the efficacy of HF, it is useful to control values of the middle-weight and low-weight molecules in blood and urine [34, 41, 57, 115, 142]. Initial values and the dynamics of these indices may serve as a signal to start therapy and assessment of the endotoxicosis stage and renal function competence, since these parameters are changing during treatment in patients with endogenous intoxication and pancreonecrosis [34]. This can be judged by the average decrease of middleweight molecule quantity by 14.3%, leukocyte index of intoxication by 24.2%, alkaline phosphatase by 4.4%, total bilirubin by 40.3%, creatinine by 33.3%, and blood amylase by 28.5% on the first day of treatment relative to the initial data. Values are normalized 5–7 days after the beginning of treatment [34]. C-reactive protein, intestinal proteins, interleukins, amylase, lipase, procalcitonin can also serve as biochemical markers demonstrating the effectiveness of continuous RRT methods [38, 44, 51, 52, 57, 62, 73, 128, 143, 144].

Thus, despite the experience gained in Russia and abroad, a unified grounded methodology and strategy of using continuous RRT by extrarenal indications in critical states, in AP in particular, has not yet been developed. Common and unified indications for the beginning of the procedure, regimens, and its completion have not been also defined. Rather often, different approaches to the application of continuous RRT are encountered in various clinics. A top priority on the agenda is discussion of important procedural issues predetermining successful application of this method such as timely initiation of the procedure, its duration with the adequate replacement rate under the control of endotoxicosis indices as well as employment of membranes with good filtration and sorption characteristics.

## Unsolved questions of using hemofiltration in patients with severe acute pancreatitis

Nowadays, the role and significance of using extracorporeal methods of detoxification, HF in particular, in patients with SAP have not been fully defined [1–4]. It is indicated in the clinical recommendations of the Russian Society of Surgeons [1] that in SAP it is recommended to use the following extracorporeal methods of detoxification: a) plasmapheresis; b) HF with the power of conviction of recommendation “D”.

It was stated in the National Guidelines on Intensive Care [4] that routine application of RRT in the absence of AKF and extrarenal indications does not influence the prognosis. In this connection, it is recommended to use hemodialysis, HF, and HDF only if signs of AKF or extrarenal indications have appeared in patients with SAP (uncorrectable metabolic acidosis, dysnatriemia, hyperthermia over 39.5°, edema of the lungs or brains.

Some researchers [98, 145] criticize these approaches to the treatment of patients with SAP noting that these recommendations point out only on the advisability of using antimediator therapy, limit administration of antibacterial therapy, and ignore methods of continuous RRT though there is enough evidence of their efficacy in the literature. The advocates of continuous RRT give examples of improved results of treating patients with SAP by one of the HF methods [34, 37, 56, 62, 64, 75, 81, 91, 105, 109, 112, 116, 117, 119, 127, 128, 133].

However, there is no unity among the authors employing HF for patients with AP on a number of key questions (whom to begin, when to begin, how to conduct, what criteria to use for the efficacy assessment, when to complete). Opinions on this point are quite different [4, 34, 37, 56, 62, 64, 75, 81, 91, 105, 109, 112, 116, 117, 119, 127, 128, 133]. One of the key questions discussed by the specialists is the determination of the time to start the HF procedure depending on the clinical picture, the process stage, disease duration, admission to the clinic, integral score assessing the status of a patient with SAP [4, 56, 91, 133].

In recent times, great attention is given in the Russian literature to the effect of intraabdominal hypertension intensity on the pathological process and prognosis of surgical diseases of abdominal cavity including AP [145–148]. Opinions are expressed concerning the advisability of laparotomy in case of the elevated intraabdominal pressure (IAP) requiring its control [145, 147, 148]. Intra-abdominal hypertension is associated with poor prognosis as it results in the perfusion impairment of abdominal organs and, perhaps, retroperitoneal space [145, 147–149]. IAP control must be a routine practice [147, 148]. Its elevation by more than 20 cm H_2_O is an indication for decompression laparotomy [145, 148].

Similar recommendations are found in the foreign literature. It is suggested to give attention to intra-abdominal hypertension, abdominal compartment syndrome, infusion therapy, and early enteral nutrition which is regulated by the materials of the World Society of the Abdominal Compartment Syndrome [150–153].

Previously, the authors [119] studying the effect of CVVH on the reduction of IAP and TNF-α level in the blood of patients with SAP and intra-abdominal hypertension, have shown that CVVH is accompanied by the decrease of TNF-α level in the blood serum and IAP. Moreover, there was found positive correlation between these two indices. Later, the same authors studied the impact of early CVVH on the reduction of IAP and IL-8 level, amylase and C-reactive protein in the blood serum of patients with SAP and abdominal compartment syndrome and established that indices of renal and liver functions improved to a greater extent in patients who underwent CVVH relative to those without CVVH administration [116].

Pupelis et al. [117] have retrospectively studied the results of 10-year application of CVVH in patients with SAP and intra-abdominal hypertension. 130 patients were included into the study. CVVH was performed in 75 patients, 55 were treated without it. In 68.5% of cases, intra-abdominal hypertension was revealed, the score according to SOFA in these patients was higher. CVVH resulted in a quicker regression of the state severity cutting the time of hospital stay.

But at the same time, the effect of HF on the enteral perfusion and IAP has not been defined until now and, therefore, the tactics of HF administration in relation to IAP has not been worked out. There is disagreement as to whether intra-abdominal hypertension is an indication for the HF beginning, what time it should be started in patients with SAP: after 48 h of organ failure or much earlier. The presented data suggest that routine monitoring of IAP should be carried out and the study of the effect of continuous RRT on the IAP changes in AP patients is to be continued.

Until now, no recommendations have been worked out concerning optimal parameters of conducting CVVH in patients in critical state, opinions differ in relation to the necessity and time to begin extracorporeal hemocorrection in SAP patients [4, 56, 67, 69, 81, 133].

There are scanty clinical investigations of the efficacy of early HF in patients with moderate degree of AP severity [75, 117]. However, owing to the appearance of the MOST concept [54, 55], one can find works on early HF application in SAP patients with encouraging results [116]. According to Aleksandrova et al. [133], HF with a replacement dose of 30 ml/kg/h in patients with early SAP restores homeostasis indices and diminishes multiple organ failure severity during a shorter period of time. The same authors have also found that continuous HF influences positively the dynamics of endogenous intoxication indices if the procedure lasts over 24 h and the replacement volume is not less than 2000 ml/h [118, 133].

At the same time, Zhang et al. [121] did not find any advantages of using early CVVH in patients with SAP and early organ failure. They employed CVVH with 2 L/h replacement volume started in the first 24 h of admission in 25 patients and compared this data with the results of treating 19 patients who did not undergo early CVVH. In the work [128], the effect of different CHVHF modes with replacement volumes of 2 and 4 L/h on the dynamics of procalcitonin inflammatory markers, TNF-α, IL-4, IL-6, IL-8, IL-10 in 86 patients with SAP and AKF has been studied. The results were evaluated after 2, 6, and 12 h of treatment and 12 h after the completion of the procedures. The reduction of the examined indices has been established to be more significant in case of using CHVHF than in the control group [128].

The authors [112] have also studied the results of CHVHF application in patients with SAP whose severity index was over 15 by APACHE II classification. Significant improvement of the state, lower risk of MOFS, shorter hospitalization period, and lethality reduction have been noted in the group in which treatment was started following the principle “as early as possible” in the first 72 h compared with the standard treatment.

Other investigators [154] compared clinical efficacy of the pulse high-volume HF and IHVHF with CVVH in patients with SAP complicated by MOFS. The study showed that in the former case the results were better and accompanied by greater reduction of biochemical marker values, better dynamics in the severity scores, smaller doses of inotropic agents.

The best results of survival due to CHVHF application are shown in the work [155]. The authors [127] also noted the decrease in lethality in patients with SAP as a result of using this HF method.

At the same time, continuous RRT procedures with small replacement volumes were not so effective. In the work of Aleksandrova et al. [133], the results of retrospective HF-method-dependent assessment of SAP course with the replacement dose of 30 ml/kg/h and more were presented. The decrease of early lethality appeared to be in the group with the replacement dose exceeding 30 ml/kg/h.

## Conclusion

As early as 2013, the authors [122] analyzed the PubMed data over the period from 1992 to 2013 in order to assess the efficacy of CVVH in patients with SAP. They found publications on various methods of HF application to 354 patients. Only two works reported significant reduction of lethality and cytokine level in blood plasma relative to the control groups. Conclusion has been made on the necessity to continue the investigations in order to find the dependence of state and outcome dynamics on the methods used (with different beginning, therapy duration, replacement rate, hemofilter types, anticoagulants, etc.).

A promising direction may be a search for optimized methods of continuous RRT: continuous veno-venous HF, hemodiafiltration, high-volume hemofiltration, continuous citrate hemodialysis, combinations with sorption technologies in patients with SAP depending on the intensity of organ dysfunctions, AKF, MOFS, septic shock in different periods of disease development [34, 56, 63, 65, 73, 79, 87, 91, 102, 103, 112, 128, 129, 154, 155].

Investigations of the applicability of APACHE II classification for assessing the state severity of SAP patients are being carried out. According to this scale, the score of 8 or more is referred to cases of SAP [1, 3, 5, 7, 12]. To assess the state dynamics, it has been also proposed to use BISAP scale and biochemical markers such as procalcitonin and C-reactive protein [1, 3, 5, 7, 12, 141, 156]. Other markers also may be suggested.

Thus, all the above said leads to the conclusion on the necessity of further investigations to find optimal indications for renal replacement therapy, search for optimal procedures and time of their initiation as well as the evaluation of their efficacy in patients with SAP.
